# Monitoring tumour response during chemo-radiotherapy: a parametric method using FDG-PET/CT images in patients with oesophageal cancer

**DOI:** 10.1186/2191-219X-4-12

**Published:** 2014-03-07

**Authors:** Pierre Vera, Bernard Dubray, Odré Palie, Irène Buvat, Sébastien Hapdey, Romain Modzelewski, Ahmed Benyoucef, Caroline Rousseau, Marc-Etienne Meyer, Stéphane Bardet, Isabelle Gardin, Frederic Di Fiore, Pierre Michel

**Affiliations:** 1 Department of Nuclear Medicine, Henri Becquerel Cancer Centre and Rouen University Hospital & QuantIF-LITIS (Equipe d'Accueil (EA) 4108–Federation Recherche (FR) National Center for Scientific Research (CNRS) 3638), Faculty of Medicine, University of Rouen, Rouen 76821, France; 2 Department of Radiation Oncology, Henri Becquerel Cancer Centre & QuantIF-LITIS (Equipe d'Accueil (EA) 4108), Faculty of Medicine, University of Rouen, Rouen 76821, France; 3 Commissariat à l'Energie Atomique (CEA)-Service Hospitalier Frédéric Joliot (SHFJ), Orsay F-75794, France; 4 Department of Nuclear Medicine, Renée Gauducheau Cancer Centre, Bd J Monod, Nantes 44850, France; 5 Department of Nuclear Medicine, Amiens University Hospital, 354 Boulevard de Beauville, Amiens 80000, France; 6 Department of Nuclear Medicine, François Baclesse Cancer Centre, 3 Avenue du Général Harris, Caen 14000, France; 7 Hepatogastroenterology Department, Digestive Oncology Unit, Rouen University Hospital, University of Rouen, Rouen 76821, France

**Keywords:** Positron emission tomography, Fluoro-deoxy-d-glucose, Oesophageal cancer, Chemo-radiotherapy, Parametric imaging

## Abstract

**Background:**

The objective of this study is to investigate the feasibility and the additional interest of a parametric imaging (PI) method to monitor the early tumour metabolic response in a prospective series of oesophageal cancer patients who underwent positron emission tomography with fluoro-2-deoxy-d-glucose (FDG-PET/CT) before and during curative-intent chemo-radiotherapy.

**Methods:**

Fifty-seven patients with squamous cell carcinoma (SCC) of the oesophagus prospectively underwent FDG-PET/CT before chemo-radiotherapy (CRT) (PET_1_) and at 21 ± 3 days after the beginning of CRT (PET_2_). The outcome was assessed at 3 months and 1 year after the completion of CRT (clinical examination, CT scan or FDG-PET/CT, biopsy). For each patient, PET_1_ and PET_2_ were registered using CT images. The 2 PET image sets were subtracted, so the voxels with significant changes in FDG uptake were identified. A model-based analysis of this graph was used to identify the tumour voxels in which significant changes occurred between the two scans and yielded indices characterising these changes (green and red clusters). Quantitative parameters were compared with clinical outcome at 3 months and at 1 year.

**Results:**

The baseline tumour FDG uptake decreased significantly at PET_2_ (*p* < 0.0001). The tumour volume significantly decreased between PET_1_ and PET_2_ (*p* < 0.02). The initial functional volume of the lesion (TV_1_) was significantly lower (*p* < 0.02) in patients in clinical response (CR) at 3 months and 1 year. The volume of the lesion during the treatment (TV_2_) was significantly lower in patients identified as in CR at 3 months (*p* < 0.03), but did not predict the outcome at 1 year. Multivariate analyses of outcome at 3 months showed that the risk of failure/death increased with younger age (*p* = 0.001), larger metabolic volume on PET_1_ (*p* = 0.009) and larger volume with decreased FDG uptake (*p* = 0.047). As for outcome at 1 year, the risk of failure/death increased with younger age (*p* = 0.006), nodal involvement (*p* = 0.08) and larger volumes with increased uptake (*p* = 0.03).

**Conclusion:**

A parametric method to assess tumour response on serial FDG-PET performed during chemo-radiotherapy was evaluated. Early metabolic changes, i.e. variations in FDG uptake, provided additional prognostic information in multivariate analyses ClinicalTrials.gov NCT 00934505.

**Trial registration:**

Current Controlled Trials ISRCTN7824458

## Background

Positron emission tomography with fluoro-2-deoxy-d-glucose (FDG-PET/CT) is commonly used in the initial staging and post-treatment follow-up of many cancer patients [1,2]. Since metabolic changes under treatment are likely to precede anatomic alterations [3-5], FDG-PET/CT is actively investigated as a way to assess tumour response to chemotherapy (CT) and radiotherapy (RT) in lymphomas [6,7], non-small cell lung [8-10], head and neck [11], and colorectal and breast cancers [12].

Oesophageal cancer is the third most frequent gastro-intestinal cancer, with a poor prognosis and high mortality rates (5-year survival rates around 4% to 10% [13]). In clinical practice, FDG-PET has a well-established role in the diagnosis and staging of oesophageal cancer [14]. Comparisons of FDG uptake before and after treatment reported a better outcome in patients with complete metabolic response [15,16]. Definitive chemo-radiotherapy (CRT) has become the first-line therapeutic standard in patients with locally advanced tumours [17,18]. At the time of diagnosis, less than 50% of these patients have potentially operable tumours [19]. The early identification (e.g. around 25 to 30 Gy) of the tumours that do not respond to CRT would suggest to go for surgery and avoid full-dose CRT toxicity. Due to the heterogeneity of the published series [20], FDG-PET/CT cannot be yet recommended in routine practice to guide the initial treatment of patients with oesophageal cancer [21], particularly in those with squamous cell carcinoma [22].

The guidelines for tumour response assessment [23] include several indices quantifying FDG uptake (standard uptake value (SUV)max, SUVmean, SUVpeak and total lesion glycolysis (TLG)) or metabolic volume (tumour longitudinal length (TL) and tumour volume (TV)) [24,25]. These indices are calculated on a regional basis, i.e. represent index values measured over the whole tumour. Complex changes in tumour uptake/volume, namely heterogeneity in tumour response, can therefore be overlooked. An automatic method using parametric imaging (PI) has been proposed to quantify FDG uptake variations in metastatic colorectal cancers [26]. The main interest of this approach is its ability to describe heterogeneous tumour response at the voxel level.

In a previous analysis of 57 patients with oesophageal squamous cell carcinoma (SCC) and recruited in a prospective study [27], we showed that the parameters derived from baseline FDG-PET were good predictors of outcome after CRT: larger tumour volume and higher SUVmax/TLG were associated to poor outcome at 3 months. Higher SUVmax values were also predictors of poor outcome at 1 year. FDG-PET performed during CRT at day 21 appeared to be of lower clinical relevance. We present a reanalysis of the same series, where the PI method was used to investigate the predictive value of the metabolic variations observed between FDG-PET/CT performed before and during curative-intent CRT. Our goal was to demonstrate that the PI method was applicable to oesophageal SCC treated with concomitant radiotherapy and chemotherapy (and not limited to colorectal cancers after chemotherapy as initially described) and that it would provide additional information to conventional clinical and FDG-PET/CT data.

## Methods

### Study design

The design of the study (RTEP3, NCT 00934505, http://www.clinicaltrials.gov/) has been previously described (see details in [27]). Briefly, patients with histological proof of oesophageal SCC and candidate to curative-intent CRT [28] were prospectively included after signing a consent form. The target sample size was 100 patients. Slow recruitment led us to close the study after the inclusion of 57 patients, an intermediary analysis showing that statistical significance would not be reached with the planned sample size.

Initial staging included oesophagoscopy with biopsies, chest and abdominal computed tomography (CT scan) and endoscopic ultrasound. Each patient underwent FDG-PET at baseline within 15 days before CRT (PET_1_) and at day 21 (± 3 days) of CRT (PET_2_). Tumour response was assessed at 3 months and 1 year after CRT with clinical investigation, CT or FDG-PET/CT, and oesophagoscopy with biopsies (if possible).

### FDG-PET imaging

The images were acquired with the arms positioned over the head and with free breathing. Six to eight bed positions per patient were acquired from the head to the upper third of the thighs. The FDG-PET scanners used were as follows: Biograph Sensation 16 (Siemens, Erlangen, Germany), Gemini (Philips, Best, The Netherlands) and Discovery LS (General Electric Medical Systems, Milwaukie, OR, USA). A specific phantom [29] was developed and used to compare and follow the quality control of the PET devices in the participating centres. For each patient, two FDG-PET scans were performed using the same machine and under the same operational conditions, i.e. the patients fasted overnight or for at least 6 h, blood glucose levels were measured before each FDG-PET/CT. A total of 4.5 MBq/kg was administered intravenously after a rest period of at least 20 min. The acquisitions had to start at 60 ± 10 min post-injection. The same post-injection delay (±5 min) was mandatory for PET_2_ during CRT. Reconstruction of the PET images was performed using ordered subset expectation maximisation (OSEM). The PET images were corrected for random coincidences, scatter and attenuation using the CT scan data.

### FDG-PET/CT analysis

All of the FDG-PET/CT images were collected in Rouen to insure homogeneous analyses.

### Quantitative analysis

The FDG-PET/CT images were analysed on a Leonardo® clinical workstation with TrueD® software (Siemens Medical Solutions, Hoffman Estates, Knoxville, TN, USA). For each patient, an experienced nuclear physician selected regions of interest (ROIs) in the most intense areas of FDG accumulation in the primary tumour on PET_1_. Any increased FDG uptake was compared with the anatomical findings from the CT scan. The presence of possible oesophagitis was defined by a moderate FDG uptake on the PET_2_, with a disappearance of uptake at 3-month and/or 1-year follow-up and the absence of recurrence at 3 months and 1 year. The tumour volume (cm^3^) was manually determined by a single nuclear medicine physician (OP) using a percentage of the SUVmax (TV_1_ for PET_1_ and TV_2_ for PET_2_). The reproducibility and the advantage of the visual determination of the functional volume by physician have been previously shown and discussed [27]. The maximum SUV was defined as the highest-activity voxel value (SUVmax_1_ for PET_1_ and SUVmax_2_ for PET_2_). The mean SUV in TV was defined as the mean tumour activity concentration (SUVmean_1_ for PET_1_ and SUVmean_2_ for PET_2_). The percentage (Δ%) of change between PET_1_ and PET_2_ was calculated as (PET_1_-PET_2_)/PET_1_ for SUVmax (Δ%SUVmax), SUVmean (Δ%SUVmean) and TV (Δ%TV).

### Parametric imaging method

The salient feature of the PI analysis is to assess the changes in metabolic activity at the voxel level in order to underline heterogeneities in tumour response [26]. The FDG-PET/CT images were transferred on a Dosisoft workstation (v 1.4, Oncoplanet, DosiSoft, Cachan, France). For each patient, a large cubic VOI was selected in the most intense area of FDG accumulation in the primary tumour and lymph nodes on the baseline (PET_1_) and mid-treatment PET images (PET_2_), by an experienced nuclear physician. PET_1_ and PET_2_ were co-registered using a rigid method, under visual control and combined with an affine method when necessary. The registration was restricted to the thorax to limit the uncertainties on the oesophagus position. PET_1_ and PET_2_ datasets were subtracted, yielding a 3D image of the VOI, with the signal in each voxel *i* being proportional to the difference in SUV: DIFF(*i*) = (SUV_2_(*i*) − SUV_1_(*i*)). Then, the voxels of DIFF were classified into four classes according to the voxel values in both the PET_1_ and DIFF datasets as follows:

Cl_1_: high SUV on PET_1_ and decreased SUV on PET_2_

Cl_2_: SUV increased between PET_1_ and PET_2_

Cl_3_: low SUV on PET_1_ with no substantial SUV changes at PET_2_ (i.e. background, etc.)

Cl_4_: voxels in which physiologic changes are not related to the tumour masses (i.e. voxels with a low SUV on PET_1_) (i.e. heart, lung disease, etc.).

Voxel classification was performed using a stochastic expectation maximisation algorithm, assuming a Gaussian mixture model (GMM) for the distribution of voxel values [26]. A parametric dataset was created from DIFF by setting the signal in voxels belonging to Cl_3_ and Cl_4_ to zero. For visualisation purpose, the voxels belonging to Cl_1_ (decreased uptake) were coded on a green colour scale, and the voxels belonging to Cl_2_ (increased uptake) were coded on a red colour scale. By definition, no stable voxel activity could be observed in the parametric volume. At the end of the process, the PI consists of one or several clusters of voxels either red (r) or green (g). The green clusters represent the part(s) of the tumours with decreased in SUV, while the red clusters represent the part(s) with increase in SUV between PET_1_ and PET_2_. The cluster volume (*V*_r_ or *V*_g_ in cm^3^) was calculated for each cluster.

### Statistical analysis

The primary endpoint was disease-free survival at 3 months and 1 year after treatment, with local/regional/distant relapse or death being considered as events. Complete response (CR) to CRT was defined as no residual tumour detected at endoscopy (negative biopsies) and without regional or distant disease on CT or FDG-PET/CT.

All statistical analyses were performed with NCSS software (version 07.1.18, Kaysville, UT, USA). As for univariate analyses, categorical variables were compared using chi-squared tests, with Yates' correction for small samples. Quantitative variables were compared using *t* tests after natural logarithm transformation to obtain Gaussian distributions. Multivariate analyses were performed using stepwise logistic regression. A threshold of *p* ≤ 0.05 was considered as statistically significant (bilateral tests).

## Results

Fifty-seven patients were prospectively included. Patients 8, 16, 25, 31 and 40 were secondarily excluded because of disease progression at the time of diagnosis. Patient 14 died during RCT. In comparison with the initial study [27], and for technical reasons related to the impossibility to use CT images in the co-registration method in the software (non joined slices), the PET_1_ of patients 42 and 45 and the PET_2_ of patients 6, 27 and 50 were not available. As a result, 46 patients were fully evaluable.

**Figure 1:**
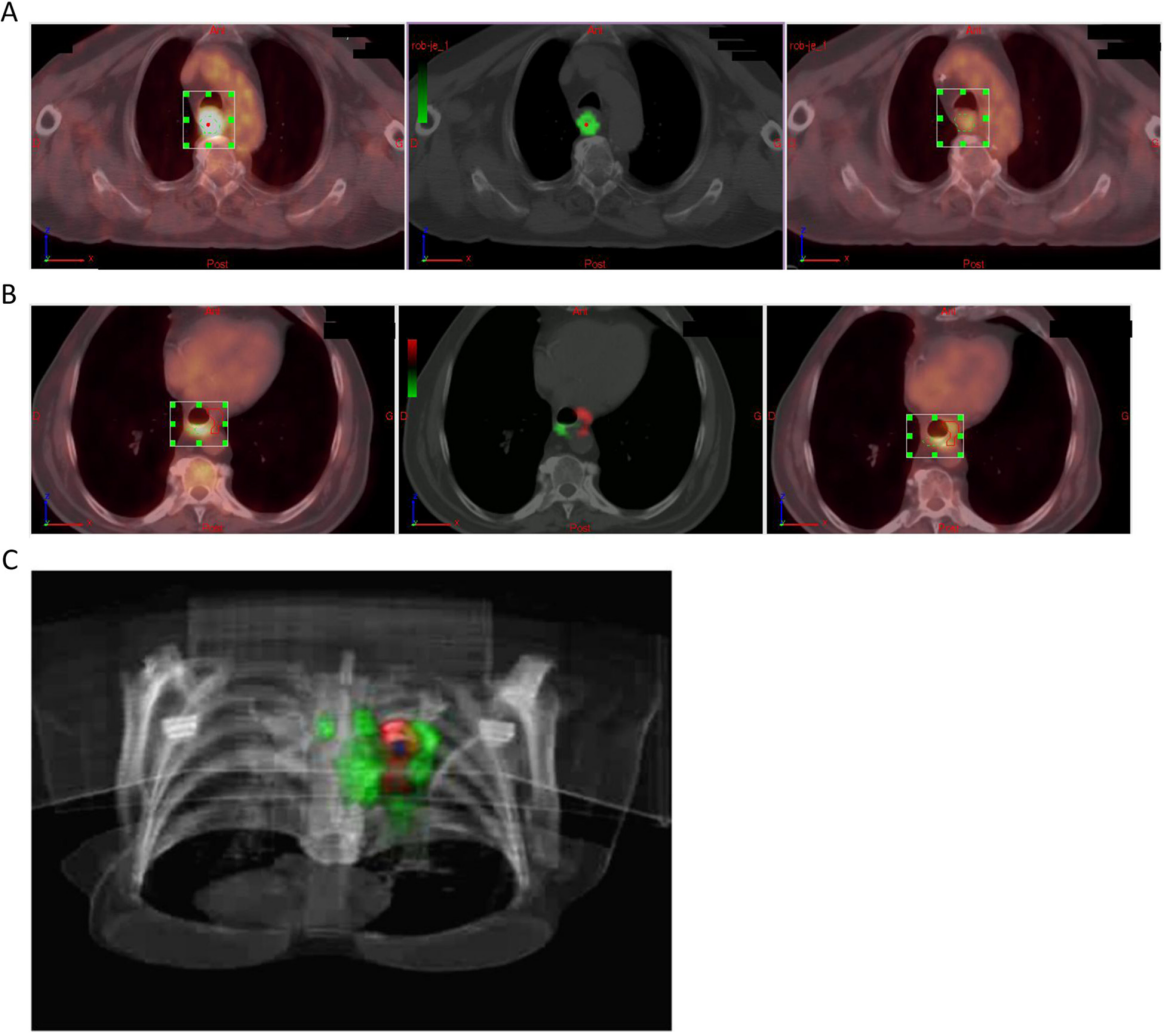
**Parametric analysis of the variations in FDG uptake before and during treatment.** Left panel: before treatment (TEP1), right panel: at day 21 during treatment (TEP_2_), middle panel: co-registration of TEP_1_ and TEP_2_. The green voxels are those in which FDG uptake has decreased between TEP_1_ and TEP_2_; the red voxels are those in which FDG uptake has increased. Voxels in which FDG uptake remained stable do not appear. **(A)** All voxels are green, indicating homogeneous decrease in FDG uptake (patient was in CR at 1, but small *V*_g_ of 6 cm^3^). **(B)** An example of spatially heterogeneous response, with green and red voxels appearing in the same tumour (patient with recurrence at 1 year). **(C)** A 3D visualisation of the PI imaging.

Table 1 shows the characteristics of the 46 evaluable patients. Five patients (11%) had cancer extending to more than one anatomical third of the oesophagus. Twenty-nine (63%) and 22 (48%) patients were alive without disease at 3 months and at 1 year, respectively.At least one green cluster was observed in all the 46 evaluable patients, demonstrating some SUV decrease between the two PET examinations. Eight patients had two green clusters, none had three green clusters, one had four green clusters, and two had five green clusters. Fifteen patients had at least one red cluster, nine had two red clusters, two had three red clusters, one had four red clusters, and one had five red clusters. The presence of both green and red clusters in the same patient illustrates the spatially heterogeneous evolution of lesions' uptake. An example is shown in Figure 1.

**Table 1:** Patients' characteristics

**Characteristics**	**Evaluable patients (46)**
Sex: F/M (%)	4 (9)/42 (91)
Age (years), mean (range)	62 (39 to 82)
WHO status: *n* (%)	
0	20 (43)
1	16 (35)
2	3 (7)
Undetermined	7 (15)
T/N stage: *n* (%)	
cT2	6 (13)
cT3	35 (76)
cT4	5 (11)
cN + ^a^	16 (35)
Tumour location (third): *n* (%)	
Upper	13 (28)
Middle	18 (39)
Lower	10 (22)
Upper + middle	3 (7)
Middle + lower	2 (4)

^a^Enlarged lymph nodes on CT scan or EUS or metabolic lymph nodes on FDG-PET/CT.

The patients who died or those without complete remission at 3 months (Table 2) were more likely to have T4 cancer, tumour extending to more than one third of the oesophagus, nodal involvement, larger metabolic volumes on PET_1_ and PET_2_, and larger green (regression) or red (progression) volumes on FDG-PET/CT parametric analysis. At 1 year (Table 3), the patients who died or those without complete remission were younger, had larger tumours (more than one third of the oesophagus and larger metabolic volume on PET1) with more frequent nodal involvement and had larger red volumes (progression).

**Table 2:** Univariate analysis according to outcome at 3 months

	**Endpoint at 3 months**		
	**CR (*n*** **= 29)**	**Failure/death (*n*** **= 17)**	***p***
Clinical factors			
Age	64.0 (59.6 to 68.3)	59.4 (53.9 to 64.9)	0.18
F/M	3/26	1/16	1.0
T2/T3/T4	2/27/0	4/8/5	0.001
1/2-site T	28/1	13/4	0.055
N0/N1	22/7	8/9	0.048
FDG-PET/CT parameters			
SUVmax_1_	12.5 (10.8 to 14.2)	15.4 (10.9 to 20.0)	0.44
TV_1_	9.7 (7.2 to 13.1)	20.6 (13.2 to 32.2)	0.004
SUVmax_2_	7.1 (5.9 to 8.2)	8.6 (6.4 to 10.9)	0.23
TV_2_	4.8 (3.3 to 6.9)	11.1 (4.6 to 13.6)	0.009
*V*_g_	3.1 (1.8 to 5.1)	5.8 (3.5 to 9.8)	0.056
*V*_r_	5.6 (4.0 to 7.9)	9.8 (6.2 to 15.5)	0.047

Quantitative variables: mean (95% confidence limits).

**Table 3:** Univariate analysis according to outcome at 1 year

	**Endpoint at 1 year**		
	**CR (*n*** **= 22)**	**Failure/death (*n*** **= 24)**	***p***
Clinical factors			
Age	66.4 (62.3 to 70.5)	58.5 (53.5 to 63.5)	0.02
F/M	3/19	1/23	0.34
T2/T3/T4	3/19/0	3/16/5	0.08
1/2-site T	22/0	19/5	0.05
N0/N1	21/1	9/15	<10^−4^
FDG-PET/CT parameters			
SUVmax_1_	10.9 (9.3 to 12.9)	13.7 (11.2 to 16.8)	0.08
TV_1_	9.0 (6.5 to 12.5)	17.8 (12.2 to 26.0)	0.007
SUVmax_2_	6.3 (5.2 to 7.6)	7.5 (6.0 to 9.3)	0.22
TV_2_	6.2 (3.7 to 10.2)	7.0 (4.5 to 10.7)	0.69
*V*_g_	3.2 (2.1 to 4.7)	5.3 (2.9 to 9.8)	0.33
*V*_r_	4.5 (3.1 to 6.6)	10.2 (7.1 to 14.5)	0.002

Quantitative variables: mean (95% confidence limits).

Multivariate analyses (Table 4) of outcome at 3 months showed that the risk of failure/death increased with younger age (*p* = 0.001), larger metabolic volume on PET_1_ (*p* = 0.009) and larger green volume (*p* = 0.047). As for outcome at 1 year, the risk of failure/death increased with younger age (*p* = 0.006), nodal involvement (*p* = 0.08) and larger red volumes (*p* = 0.03).

**Table 4:** Multivariate analysis of outcome at 3 months and 1 year (logistic regression)

**Endpoint**	**Covariates**	**Odds ratio (95% CI)**	***p***	**Correctly classified**	**AUC ROC**
3 months	Age	0.94 (0.90 to 0.98)	0.001	76%	0.84
	Ln(TV_1_)	2.93 (1.31 to 6.60)	0.009		
	Ln(1 + *V*_g_)	2.23 (1.01 to 4.96)	0.047		
1 year	Age	0.95 (0.92 to 0.99)	0.006	78%	0.89
	N+	20.9 (2.24 to 195)	0.008		
	Ln(*V*_r_)	3.08 (1.12 to 8.46)	0.03		

Natural logarithm (Ln) transformations were used to obtain Gaussian distributions for quantitative variables. 95% CL, 95% confidence limits; AUC ROC, area under receiver operating characteristics curve.

## Discussion

In this prospective, multicentre study, an automated parametric method [26] was successfully used to investigate the prognostic value of variations in FDG uptake during curative-intent RCT in oesophageal squamous cell carcinoma. The co-registration of sequential PET examinations allows a voxel-based analysis, as a way to investigate spatial variability in tumour response to treatment. In multivariate analyses, a larger volume of red voxels, i.e. with increased uptake between baseline PET and PET performed at day 21, was significantly associated with a greater probability of treatment failure/death at 1 year.

Our results were obtained in a homogeneous population of patients with oesophageal squamous cell carcinoma. Oesophageal squamous cell carcinoma is very sensitive to CRT [30]. During CRT, SUVmax, SUVmean and TV decreased sharply (Table 2). The SUV value depends on several parameters: delay between injection and acquisition [31], noise level, spatial resolution in the reconstructed images, and region selected to estimate the SUV [32]. SUV measurements have been shown to vary between centres [33]; thus, multicentre studies require rigorous standardisation of the FDG-PET procedures [34]. In the present study, all of the paired FDG-PET/CTs for a given patient were performed in the same department, avoiding inter-centre variability. The cross-calibration of the participating centres was assessed in a previous clinical study [35]. The performance and quality control of the PET/CT devices were monitored by using a specific phantom developed by our group [29], and the post-injection delay was kept constant to facilitate inter-patient and inter-centre comparison. All quantitative analyses were performed in Rouen on the same workstation by one nuclear medicine physician. We relied on an experienced nuclear medicine physician to delineate the tumour metabolic volume (TV) since low FDG uptake on images acquired during treatment limits the use of automatic segmentation methods [8]. Acute inflammatory reactions, as reported in head and neck cancer [36], may also hamper the analyses of PET images during treatment. Oesophagitis was present in 9 out of 46 (19%) patients, and 3 of 46 had a nasogastric feeding tube.

In a previous analysis of the same group of patients [27], smaller TVs at baseline (PET_1_) and at day 21 (PET_2_) were associated to higher probabilities of response at 3 months, as already reported by other investigators [25,37,38]. However, we failed to demonstrate a prognostic value for Δ%SUVmax, Δ%SUVmean or Δ%TV, showing similar evolution of these quantitative parameters whatever the tumour response to treatment. These indices are calculated over the whole tumour and do not address spatial variations of response to treatment within the tumour. In the present report, we aimed at investigating intra-tumour heterogeneity in FDG uptake as an early measure of response to treatment, our results being in line with the encouraging ones published in oesophageal (mixing SCC and adenocarcinomas), cervical and head and neck cancers [39,40]. In both analyses, the patients with CR (either at 3 months or at 1 year) were older than the ones who died or whose tumour relapsed. These differences were statistically significant at 3 months and 1 year in the present analysis, varying from our original report [27], where age was a predictor of outcome only at 1 year and not at 3 months, possibly as a consequence of the exclusion of two patients and/or the introduction of new variables (*V*_g_ and *V*_r_) in the logistic model.

Our aim was to characterise the variations in FDG uptake at the voxel level. The parameters were extracted from an image set combining the FDG-PET/CTs performed before and during CRT. This procedure is similar to the use of ictal and interictal perfusion single-photon emission computed tomography (SPECT) in the SISCOM procedure [41]. Such an approach critically relies on registration accuracy. Our FDG-PET/CTs were co-registered over the thorax region using a rigid method, then visually inspected and, if necessary, re-registered with an affine registration method. The repeatability of the method has been previously validated, as well as the impact of misregistration on the generation of green and red clusters [26]. The classification of the voxels according to the differences in uptake at PET_1_ and PET_2_ and their position as regards the CT tumour volume were visually checked. Some voxels obviously belonging to the heart or related to oesophagitis had to be manually re-classified. Our previous analysis [27] failed to support FDG uptake or metabolic volume measured on PET_2_ as prognostic/predictive indices. In the present study, a predictive value of *V*_r_ and *V*_g_ (including Ln(*V*_r_) or Ln(1 + *V*_g_)) was demonstrated in multivariate analyses, suggesting that PET_2_ could be of clinical interest.

The main feature of the present parametric method presented here is the quantification of tumour response to treatment at the voxel level, possibly more informative than a single value (e.g. SUVmax) calculated over the whole functional volume. Our method seems attractive when monitoring tumour response on serial FDG-PET/CT as oesophageal cancers are hardly visible on the CT images. The present method could become a valuable tool to quantify dissociated responses on multiple tumour sites, e.g. primary tumour and metastatic nodes, provided that all the regions of interest are selected. Multivariate analyses showed that the parametric method added some predictive value to more conventional variables. The association between larger green volumes (i.e. decreased uptake between PET_1_ and PET_2_) and worse outcome at 3 months appears to be counter-intuitive. Our data suggest that large initial tumour volumes are the most likely (1) to shrink during treatment (explaining the large absolute green volumes) and (2) to shrink only partially, so persistent activity is still visible on PET_2_. For example, if a 50-cm^3^ functional volume at PET_1_ decreased to 30 cm^3^ at PET_2_ (i.e. a major response), a large green volume (20 cm^3^) would be anyway associated to a bad outcome related to the large initial volume. In contrast, a small green volume (e.g. 2 cm^3^) would be measured on a 5-cm^3^ initial functional volume with the response of similar amplitude, i.e. a 3-cm^3^ residual volume at PET_2_, also associated to treatment failure despite a relatively small initial volume. Statistically speaking, our limited number of patients precluded us to test for interaction between initial and green volumes. The omission of the green volume from the multivariate analyses did not alter the proportion of correctly classified patients (76% (3 months) and 78% (1 year)), but slightly decreased the area under the ROC curve (from 0.84 to 0.79).

A limitation of the present study is the relatively small number of evaluable patients. The planned sample size was not reached due to accrual slower than anticipated [27]. We could anyway demonstrate that 46 patients with oesophageal SCC could be prospectively recruited in a multicenter setting, all investigators abiding to strict acquisition procedures and analysed together using an innovative approach. The clinical value of FDG-PET/CT during radiotherapy deserves further validation, possibly using the method presented here.

## Conclusion

This prospective, multicentre study performed in patients with squamous cell oesophageal cancer evaluated a parametric method to monitor heterogeneous tumour response patterns on serial FDG-PET/CT images acquired during radiotherapy. We demonstrate its feasibility and ability to characterise early metabolic changes and suggest that it provides added prognostic information to conventional variables such as SUVmax and TV.

## Competing interests

The authors declare that they have no competing interests.

## Authors’ contributions

PV and PM designed the study. PV, BD and OP did the data analysis and writing of the paper. IB, IG, SH and RM developed the method. PM, AB, CR, M-EM, FDF and SB recruited the patients. All authors read and approved the final manuscript.
